# Soil Organic Carbon and Total Nitrogen Gains in an Old Growth Deciduous Forest in Germany

**DOI:** 10.1371/journal.pone.0089364

**Published:** 2014-02-20

**Authors:** Marion Schrumpf, Klaus Kaiser, Ernst-Detlef Schulze

**Affiliations:** 1 Max Planck Institute for Biogeochemistry, Hans-Knöll-Straße 10, 07745 Jena, Germany; 2 Soil Sciences, Martin Luther University Halle-Wittenberg, 06120 Halle (Saale), Germany; Catalan Institute for Water Research (ICRA), Spain

## Abstract

Temperate forests are assumed to be organic carbon (OC) sinks, either because of biomass increases upon elevated CO_2_ in the atmosphere and large nitrogen deposition, or due to their age structure. Respective changes in soil OC and total nitrogen (TN) storage have rarely been proven. We analysed OC, TN, and bulk densities of 100 soil cores sampled along a regular grid in an old-growth deciduous forest at the Hainich National Park, Germany, in 2004 and again in 2009. Concentrations of OC and TN increased significantly from 2004 to 2009, mostly in the upper 0–20 cm of the mineral soil. Changes in the fine earth masses per soil volume impeded the detection of OC changes based on fixed soil volumes. When calculated on average fine earth masses, OC stocks increased by 323±146 g m^−2^ and TN stocks by 39±10 g m^−2^ at 0–20 cm soil depth from 2004 to 2009, giving average annual accumulation rates of 65±29 g OC m^−2^ yr^−1^ and 7.8±2 g N m^−2^ yr^−1^. Accumulation rates were largest in the upper part of the B horizon. Regional increases in forest biomass, either due to recovery of forest biomass from previous forest management or to fertilization by elevated CO_2_ and N deposition, are likely causes for the gains in soil OC and TN. As TN increased stronger (1.3% yr^−1^ of existing stocks) than OC (0.9% yr^−1^), the OC-to-TN ratios declined significantly. Results of regression analyses between changes in OC and TN stocks suggest that at no change in OC, still 3.8 g TN m^−2^ yr^−1^ accumulated. Potential causes for the increase in TN in excess to OC are fixation of inorganic N by the clay-rich soil or changes in microbial communities. The increase in soil OC corresponded on average to 6–13% of the estimated increase in net biome productivity.

## Introduction

Terrestrial ecosystems are facing a number of environmental shifts, such as rising CO_2_ in the atmosphere, increasing temperature, changes in precipitation, increasing nitrogen deposition, or increased frequency of extreme weather events. These factors can affect ecosystem carbon storage and thereby feed back to climate change [1], [2]. A number of ecosystem manipulation experiments have been carried out to determine effects of changing environmental conditions on ecosystem processes [3]. It is, however, not possible to simulate environmental changes of entire forest ecosystems, and often only one factor is modified in controlled experiments while many changes occur simultaneously under real-world conditions. Determination of net responses of ecosystems to environmental changes, therefore, requires monitoring of ecosystem processes under natural field conditions.

Ballentyne et al. [4] showed that the net CO_2_ uptake by terrestrial and aquatic ecosystems doubled during the last 50 years but it is still unresolved where the carbon goes [4], [5]. Forests seem to be important terrestrial carbon sinks [6]–[8]. Besides environmental factors, long-term changes in forest cover and management need to be considered as possible causes for forest organic carbon (OC) accumulation or depletion [9]. Only few results on repeated forest soil OC inventories exist to date [10]. These predominantly report increases in forest soil OC (Table 1), rarely declines (i.e., Belgium region Wallonia, [11] England and Wales, [12]). Many repeated soil inventories did not determine bulk densities directly. Instead, these were derived from pedotransfer functions, an approach that increases the uncertainty, especially if bulk density is not constant over time [13]–[15]. Accordingly, additional repeated inventories of forest soil OC stocks that include determination of all contributing variables are needed.

**Table 1:** Measured and modelled changes in soil C stocks in temperate forests.

Location		Soil depth	Change	Years	ref
		cm	g C m^−2^ yr^−1^		
Europe	Region	0–100	60	1990–1999	[8]
			46	2000–2007	
Belgium	Region	0–30	68	1960–2000	[50]
Germany	Region	0–30	50	1990–2006	[51]
Sweden	Region	humus layer	25	1961–2002	[52]
Belgium	Region	0–30	−23	1950–2006	[11]
China	Plot	0–20	61	1979–2003	[14]
Germany	Plot	0–30	21 to 40	1974–2004	[9]
USA	Plot	0–10	−11 to +6	1976–2006	[30]
Germany	Plot	0–60	27–65	2004–2009	This study

Following stoichiometric principles, gains in soil OC will go along with increases in other elements including nitrogen (N) [16]. The combined changes in soil OC and N can give hints on sources of accumulating organic matter as input of new plant litter, for example, has larger OC-to-N ratios than bulk soil organic matter. Another factor potentially affecting the soil OC-to-N ratio is N deposition. It is assumed to contribute to enhanced plant growth under elevated CO_2_, leading to increased organic matter input and storage in soil [17]. Only part of the N added to ecosystems is taken up by plants, and soils often play the more important role in ecosystem N retention [18], [19]. Thus, long-term N deposition should lead to N accumulation in soil if no N is lost due to leaching or denitrification. Although it is well known that C and N cycles are closely linked and the fate of deposited N is still not resolved, the focus of repeated soil inventories is often on OC only, while changes in N are not reported.

For the old-growth deciduous forest in the Hainich National Park, Germany, eddy covariance analyses indicated the site as a strong carbon sink of almost 500 g m^−2^ yr^−1^ [20], but Kutsch et al. [21] showed that some of that was erroneous due to advection. Based on soil carbon budgets calculated as difference between input and heterotrophic respiration, Kutsch et al. [22] concluded that the soil can only be a small net carbon sink of 1 to 35 g m^−2^ yr^−1^. Tefs and Gleixner [23], on the other hand, observed soil gains of 164 g OC m^−2^ yr^−1^ and 32 g TN m^−2^ yr^−1^ between 2000 and 2004, using a repeated soil inventory. However, the Pürckhauer soil corer they used for collecting samples is highly prone to compaction due to its small diameter, which hampers sampling by depth increments. Average bulk densities obtained in another soil inventory had to be used to convert concentration differences into fluxes, which added much uncertainty to the results. The present study aimed at verifying the previous results by using an improved approach with combined determination of OC, TN, and soil masses at 100 paired soil cores down to 60 cm soil depth, sampled in 2004 and again in 2009. We hypothesize that (1) increased forest litter input leads to detectable gains in soil OC stocks after 5 years, and (2) continued N deposition results in soil N accumulation in excess to what is required according to stoichiometric principles to support OC gains.

## Materials and Methods

### Site description

The study site is located in the “Hainich National Park” in central Germany (51°04′46″N, 10°27′08″E, 440 m a.s.l.). Permission for soil sampling was granted by the headquarters of the park represented by Manfred Großmann. For detailed description of the study site, see Knohl et al. [20]. Mean annual rainfall at the eddy covariance tower site is 765 mm (range between 540 to 1050 mm from the year 2000 till 2012), and mean annual air temperature is8.3°C (6.9 to 8.8°C in the same period) (Olaf Kolle, personal communication). The site is under an old-growth mixed beech forest (65% *Fagus sylvatica*, 25% *Fraxinus excelsior*, 7% *Acer pseudoplatanus* and *A. platanoides*). Trees have a wide range of ages, with a maximum of 250 years. Maximum tree height varies between 30 and 35 m, and the leaf area index is 4.8 m^2^ m^−2^. Large amounts of standing dead wood and coarse woody debris are characteristic of this semi-natural forest, which was taken out of management about 60 years ago. The ground vegetation is dominated by geophytes and hemikryptophytes and was classified as a *Hordelymo-Fagetum* [24]. The substrate for soil formation is Triassic limestone, overlain by a Pleistocene loess layer of varying thickness (10–50 cm). Soils are classified as Eutric Cambisols [25]. The litter layer is mull-type and indicates high biological activity and intensive bioturbation.

### Soil sampling

A regular grid with 30 m spacing was projected over the main footprint area (24 ha) of the eddy covariance tower, and 100 sampling points were randomly selected at grid points, marked with wooden poles having metal labels on top, and sampled in March 2004. In March 2009, new samples were taken 1.5 m north of the original sampling point. At each sampling, the litter layer was collected using a metal frame of 25 cm side length. Corers with an inner diameter of 8.7 (2004) and 8.3 cm (2009, both from Eijkelkamp Agrisearch Equipment BV, Giesbeek, The Netherlands) were used for mineral soil sampling. Closed corers were driven into soil with a motor hammer (Cobra Combi, Atlas Copco AB, Nacka, Sweden) and opened afterwards to obtain the intact soil cores. The depth of the borehole and the length of the extracted core were measured, and compared for estimation of soil compaction during coring. The average deviation between core length and hole was 0.3 cm. Soil cores were cut into 7 segments to obtain samples from 0–5, 5–10, 10–20, 20–30, 30–40, 40–50, and 50–60 cm depth. At some places, it was not possible to reach a depth of 60 cm because of stones. In such cases, a second attempt was made at 1 m distance. If necessary, the procedure was repeated a third time and the longest of the three cores used.

### Sample treatment and analyses

Soil samples were stored at 4°C prior to processing. Coarse stones of a diameter >4 mm and roots of a diameter >1 mm were removed from the samples prior to drying at 40°C. Stone and root samples were air-dried separately. Then, soil samples were sieved to <2 mm. Particles >2 mm were combined with the coarse stones. Dry weights of roots and combined stone fractions were determined. Total C and N concentrations in <2 mm soil separates were determined using dry combustion at 1100°C (VarioMax, Elementar Analysensysteme GmbH, Hanau, Germany). Carbonate C was determined after dry combustion of the samples heated in a muffle furnace at 450°C for 16 h. Organic C was calculated as the difference between total and carbonate C. Litter layer samples were dried at 70°C, shredded, and a subsample further homogenized using a ball mill. Total C and N concentrations were determined using dry combustion (Vario EL II, Elementar Analysensysteme GmbH, Hanau, Germany). Nitrogen concentrations reflect total (organic plus inorganic) N (TN). Bulk density was determined based on the dry mass of total soil material of each depth increment. Accordingly, bulk density, fine earth material (<2 mm), and OC concentrations were measured on the same samples.

### Statistics and calculations

Results are presented as means ± standard error unless indicated differently. Changes in C stocks can be calculated either for a fixed soil volume or a fixed mass of soil. Organic C and TN stocks for fixed soil volumes of soil layers were calculated based on bulk density, the relative contribution of fine earth material (soil <2 mm) to total soil mass, layer thickness, and element concentration [10]. Calculation of stocks for equivalent soil masses per area was carried out as described by Ellert and Bettany [26]. It is based on cumulative fine earth masses per area of the soil layers of each soil core (kg m^−2^). We used the average fine earth masses of each depth increment of the 2004 sampling as reference soil mass for OC stocks in both years [10]. For the calculation of OC stocks, soil material from the uppermost and underlying soil layers is cumulatively added until the desired reference soil mass is reached. Organic C stocks are then calculated by multiplying the soil mass of each included layer with the corresponding OC concentration and adding the products up to achieve total amounts of OC per area for the respective reference soil masses. We did not determine the OC concentration of the stones. Therefore, we used the fine earth mass instead of the total soil mass as a reference.

Kolmogorov-Smirnov tests were used to test the datasets for normal distribution. To test for significant changes between the sampling years, paired-sample t-tests were conducted when differences between pairs were normally distributed. Otherwise, U-tests (Wilcoxon) were performed. To determine relations between variables, Pearson correlation coefficients were calculated and tested for significance with a two-tailed t-test (p<0.05, or p<0.01, as given in figures). Statistical analyses were performed using the software package SPSS 16.0 for Windows.

## Results

Concentrations and stocks of OC and TN in 2004 [10] were significantly correlated with those in 2009 for all depth increments, indicating soil organic matter concentrations to be spatially dependent (Table 2). The closer spatial correlation of OC and TN stocks in deeper than in upper soil layers can be explained by the closer spatial dependence of the stones, and accordingly, the fine earth contents in deeper than in upper soil layers (Table 2). At 0–60 cm soil depth, the 2004 OC stocks explained 33% of the variance in stocks observed in 2009, whereas at 30–60 cm, the explained variance was 68%. The location dependence of soil OC stocks demonstrates the validity of the used paired sampling approach.

**Table 2:** Correlation coefficients for significant (p<0.05) relations among bulk density (BD), fine earth mass per m^−2^ (FE), stone content (stone), water content (WC), OC concentration (OC), total nitrogen concentration (TN), OC-to-TN ratio (CN), OC stock (OCst), and TN stock (TNst) for paired samples taken in 2004 and 2009.

Soil depth	BD	FE	Stone	WC	OC	TN	CN	OCst	TNst
cm									
0–5	ns	0.21	ns	ns	0.45	0.52	0.38	0.28	0.23
5–10	0.21	0.28	0.23	0.25	0.39	0.46	0.40	0.30	0.35
10–20	0.31	ns	0.35	0.44	0.34	0.39	0.26	0.33	0.33
20–30	0.38	0.42	0.33	0.35	0.51	0.58	ns	0.44	0.44
30–40	ns	0.46	0.46	0.42	0.48	0.46	0.22	0.54	0.57
40–50	ns	0.44	0.55	ns	0.65	0.58	0.39	0.72	0.65
50–60	ns	0.41	0.38	ns	0.65	0.50	ns	0.71	0.65

ns: not significant.

**Figure 1:**
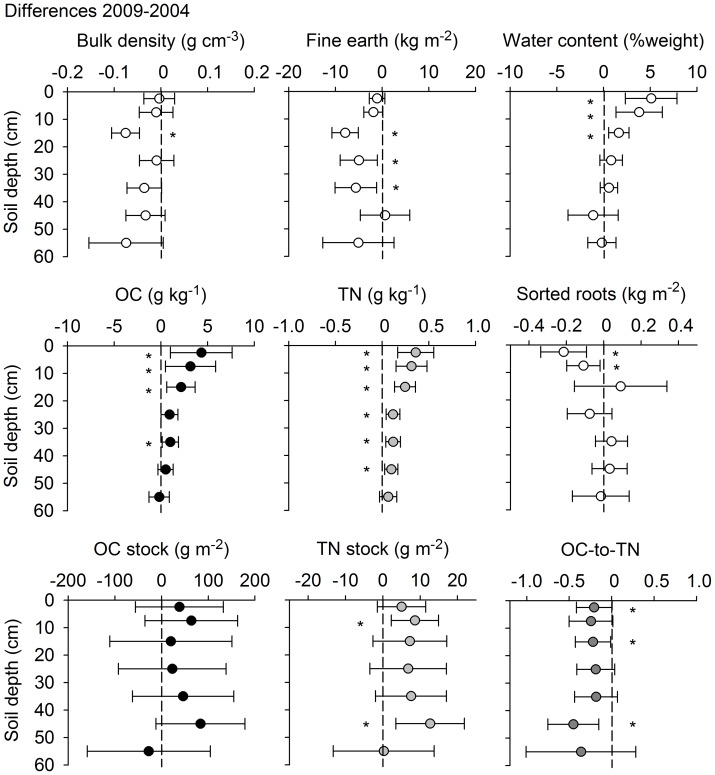
Average difference between pairs of soil cores taken in 2004 and 2009. Samples were taken for seven depth increments. Positive values indicate gains or increases with time. Error bars show 5–95% confidence intervals; asterisks indicate changes significantly different from zero.

A number of variables differed between 2004 and 2009 (Fig. 1, Table S1 in Information S1). The litter layer held significantly more OC in 2009 than in 2004 (121±24 g m^−2^ difference) and also the OC-to-TN ratio of the litter was larger in 2009 than in 2004 (by 6.4±1.8, data not shown). Concentrations of OC in the mineral soil were 7 to 9% larger in 2009 than in 2004, with differences being significant for the 0–5, 5–10, 10–20, and 30–40 cm depth layers. Increases in TN concentrations were even larger (8 to 12%) and significant throughout the profile down to 50 cm depth. The OC-to-TN ratio decreased significantly for the 0–5, 10–20, and 40–50 cm layers. Bulk densities and fine earth contents were greater in 2004 than in 2009 and negatively correlated with OC concentrations and with water contents (Fig. 1, Table in in Information S1). With OC and TN concentrations increasing and the fine earth amounts decreasing, there was no significant net change in OC stocks for individual layers. Only stocks of TN were significantly larger in the 5–10 and 40–50 cm layers.

**Figure 2:**
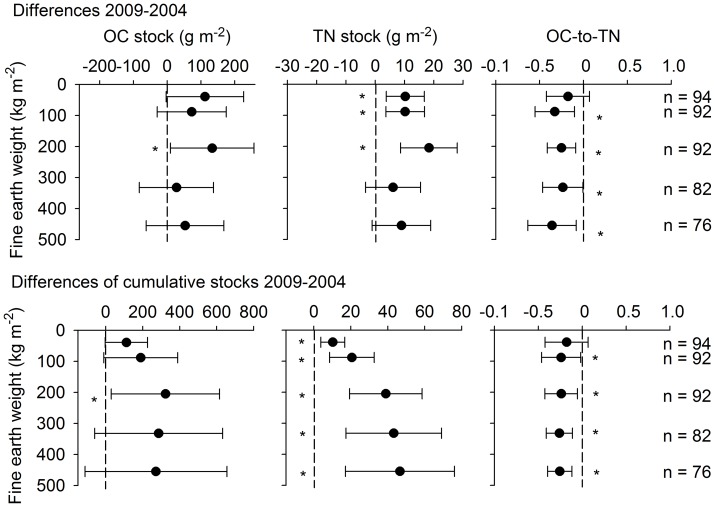
Differences in carbon and nitrogen stocks between 2004 and 2009, normalized to average soil masses in 2004. Therefore, the y-axis presents increasing cumulative fine earth masses for each depth increment of the 2004 sampling with soil depth. Upper graphs show the normalized differences of paired soil samples for each depth increment; lower graphs show the normalized differences of cumulative stocks. Positive values indicate gains or increases with time. Error bars show 5–95% confidence intervals; asterisks indicate changes significantly different from zero.

We used the average soil mass per depth increment in 2004 as reference mass for each layer when calculating OC stocks based on equal soil masses. OC stocks of equal soil masses differed significantly for the layer corresponding to 10–20 cm soil depth in 2004 (133±62 g OC m^−2^) and for the cumulative stock of the soil mass in 0–20 cm (323±146 g OC m^−2^) (Fig. 2, Table S3 in Information S1). Cumulative OC stocks did not change significantly from 2004 to 2009 when soil masses from deeper soil layers were included (Fig. 2). The largest differences in TN were also within 0–20 cm depth (39±10 g TN m^−2^), though, unlike OC, differences were significant for all individual layers to 20 cm depth when normalized to average soil masses obtained in 2004 (Fig. 2). Cumulative N stocks increased slightly below 20 cm depth from 39 to 47 g TN m^−2^. Accordingly, the OC-to-TN ratio calculated for OC and TN in fixed soil masses was significantly lower in 2009 than in 2004 below the uppermost soil layer. The average OC-to-TN ratios of the changes were 11 for the 0–5 cm, and ∼7 for the 5–10 cm, and 10–20 cm equivalents, and smaller than those of the volume-based changes calculated for the same soil layers (12, 10, and 9 for the 0–5, 5–10, and 10–20 cm layers, respectively).

**Figure 3:**
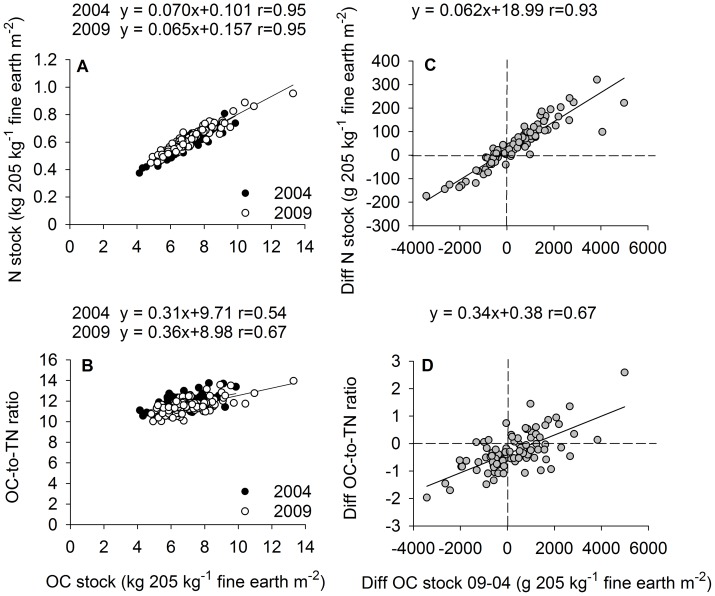
Relations between organic carbon (OC) and total nitrogen (TN). Graph A: correlations between cumulative OC and TN stocks for 205 kg fine earth m^−2^, corresponding to the average amount of fine earth per m^2^ in the 0–20 cm soil layer in 2004, for the years 2004 and 2009; Graph B: correlations between cumulative OC stocks and OC-to-TN ratios; Graph C: regression between differences between sampling times (2009–2004) for OC and TN stocks of sample pairs; Graph D: correlations between respective differences of paired samples between 2004 and 2009.

Concentrations as well as stocks of OC and TN were strongly correlated in both years and at all soil depths (Fig. 3A, Table S2 in in Information S1). The OC concentrations and stocks were positively correlated to OC-to-TN ratios in both years and at all soil depths (Fig. 3 B, Table in in Information S1). When plotting changes in stocks of OC and TN between 2004 and 2009 (Figure 3C+D) for 205 kg fine earth m^−2^ (corresponding to the average fine earth amount per m^2^ in 0 to 20 cm depth in 2004), the regression indicates that with no change in OC, TN still increased by 19±4 g m^−2^. This is gives an increase in N of 3.8±0.8 g m^−2^ yr^−1^ in excess of OC.

## Discussion

In accordance with our hypothesis, it was possible to detect statistically significant increases in soil OC and TN concentrations within 5 years. However, the more relevant detection of changes in stocks was more difficult as similar to previous studies [13], [14], our results showed that bulk densities were not constant over time. This could be due to variations in OC and water contents, or because of systematic sampling errors (e.g., compaction during coring). That highlights the necessity of determining OC and bulk density in the same inventory and to calculate stock changes based on equivalent soil masses rather than for defined soil depths [27].

Potential errors in the determination of bulk densities might have been smaller with samples taken from soil pits instead by a soil core. However, using average bulk densities from soil pits and only OC concentrations from a corer would not necessarily result in more precise estimates of soil OC stocks and stock changes due to the small scale spatial variation of both variables and their negative correlation. Calculation of OC stocks based on equal soil masses alleviates some of the problems associated with variable bulk densities. It is, however, not possible to sample correctly by soil mass. Even with bulk density determined first and defining sampling depth for determination of comparable OC concentrations based on these results, small scale spatial variations of bulk densities still hamper correct sampling, especially in stone-rich soils.

Another potential problem of soil coring is the carry-over of soil material along the soil core while drilling. This cannot be fully avoided but the moist soil cores had a stable structure, and so contamination was restricted to just the immediate layer in contact with the corer wall. Given the large diameter of the soil core (8.5 cm), effects on total soil mass or mass of C per layer were minor. Differences in carry-over of material alongside the soil core between sampling years would affect deeper layers stronger than upper soil layers, however, we observed largest increases in C concentrations in the upper horizons. There were no indications of more material being transported downwards in one year than another.

Larger amounts of roots sorted out in 2004 than in 2009 could have created an artificial increase in OC concentrations at 0–10 cm, but not at 10–20 cm depth (Fig. 1). Root picking was done following the same protocol in both years, thus, differences in roots likely are due to other causes. However, soil sampling in early spring 2004 was two weeks later and took two weeks longer than in 2009, so plants like wood garlic (*Allium ursinum*) and dog's mercury (*Mercurialis perennis*) already covered the ground, whereas they just started germination during the 2009 sampling. Accordingly, living root masses in 0–10 cm probably differed between sampling years.

Later sampling in 2004 could have also caused the smaller OC concentrations and OC-to-TN ratios of the litter layer, which indicate that litter in 2004 was already in a more advanced stage of decomposition than in 2009. The lack of an Oa horizon and the thin, discontinuous Oe horizon suggest no long-term OC accumulation in the litter layer at the study site. Therefore, we focussed the discussion on the mineral soil.

Increases in soil OC were most pronounced in the uppermost 0–20 cm of the mineral soil. Significant changes of stocks were observed for the equivalent soil mass in the 10–20 cm layer (27±12 g OC m^−2^ yr^−1^), and for the equivalent soil mass of in the entire 0–20 cm layer (65±29 g OC m^−2^ yr^−1^). These are in the same range of OC changes observed for other temperate forest sites (Table 1).

The 10–20 cm depth corresponds to the upper part of the B horizon. Gains of OC in deeper soil layers may reflect increases in root litter, and/or downward OC transport by bioturbation or with percolating soil water. Turnover times of OC in subsoils are usually longer than in topsoils, possibly due to strong stabilization of OC by association with minerals [28], [29]. Since the organic matter loading of minerals at 10–20 cm is less than that at the 0–5 cm [29], subsoils still offer more potential binding sites for OC at mineral surfaces than topsoil layers. So, long-term stabilization of additional OC in subsoil horizons seems possible.

Positive correlations between OC and the OC-to-TN ratio in the entire set of samples suggest that more input of new, litter-derived organic matter with large OC-to-TN ratios promotes larger OC concentrations at the study site. Similarly, increases in OC were positively correlated to increases of the OC-to-TN ratio, a relation that was also observed in the repeated forest soil inventory conducted by Kiser et al. [30].

The Hainich forest was intensively managed as coppice and forest grazing was practiced from the 16^th^ to the 19^th^ century [31], [32]. By the end of the 19^th^ century, the forest area was converted to selection management and the standing biomass increased from approximately 100 m^3^ ha^−1^ to 250 m^3^ ha^−1^ by the middle of the 20^th^ century [32]. Currently, the timber volume at the study site is about 540 m^3^ ha^−1^ [31]. This is still less than the observed maximum of 1000 m^3^ ha^−1^ in the Hainich region [33]. Accordingly, forest growth as a result of recovery from former coppice management could contribute to the observed soil OC gains.

Nitrogen deposition or fertilization is assumed to enhance soil OC storage by causing reduced decomposition rates as well as increased litter input [34]–[40]. Estimated N deposition rates in the Hainich region were between 1.4 (M. Mund, personal communication) and 2.1 g m^−2^ yr^−1^ [41].

Forest fertilization experiments suggest an average N use efficiency of 20 to 25 kg C kg^−1^ N for trees and 10 to 25 kg C kg^−1^ N for forest soils [42]. Assuming an annual input between 1.4 and 3.8 g N m^−2^ yr^−1^ (Fig. 3C), N additions could explain a significant portion of the observed soil OC gains (14 to 95 g m^−2^ yr^−1^).

Repeated forest inventories in the core area of the Hainich National Park revealed an increase in OC stocks from the year 2000 to 2007 with standing wood volume [33]. Observed changes correspond to an annual increase of approximately 450 g OC m^−2^ yr^−1^ or by 2.1% [33]. If this applies also to the study site, soil OC gains would be around 10% of aboveground increases. Litter trap studies at the Hainich tower site indicate an increase in annual litter fall of approximately 10 g OC m^−2^ yr^−1^ or by 2% from 2000 to 2007, further supporting the idea of increased forest production [22]. The observed change in soil OC corresponds on average to 0.9% yr^−1^ of existing stocks in the 0–20 cm soil layer, or 6 to 13% of the estimated sum of change in soil and biomass C (Net Biome Production, NBP). For the Hainich forest, the observed soil OC gain would therefore be less than half the accumulation rate of 29±15% of NBP assigned to forest soils by Luyssaert et al. [7].

Microbial biomass C (C_mic_) usually increases with soil OC [43], [44], thus, larger microbial biomass of low OC-to-TN ratios could compensate for some of the large OC-to-TN ratios of newly added litter in bulk samples. In order to assess the potential maximal impact of microbial biomass changes for changes in stocks, we assumed C_mic_ in 2004 to be 2% of the OC at 0–10 cm depth [45], and 50% larger in 2009 than in 2004. Such an increase in microbial biomass would explain OC gains of 8.3 g m^−2^ yr^−1^ and N gains of 1.0 g m^−2^ yr^−1^ (assuming an average OC-to-TN ratio of 8.6 for soil microorganisms; [46]). Given that large changes occurred at 10–20 cm soil depth, where microbial biomass is typically smaller, and that soils were sampled later in spring in 2004, when microbial biomass was probably already larger, changes in microbial biomass alone cannot explain the observed increases in soil OC and TN. Also changes in the microbial community structure, e.g., a decrease in the fungi-to-bacteria ratio in response to N input [34], could have contributed to declining OC-to-TN ratios, but this cannot be tested with the available data.

The declining OC-to-TN ratios show that soils at the Hainich site are currently an even stronger sink of TN than of OC. Deposition and accumulation of inorganic N is one possible explanation. The observed increase in soil N exceeded estimated deposition rates for the study site. As the OC-to-TN ratio in the litter layer did not decline, deposited N accumulated preferentially in the mineral soil, which is in line with other studies showing forest soils to be more effective sinks of added N than the vegetation [47]. Also, mineralization of soil organic matter results in losses of OC as CO_2_, while some of the released ammonium can be retained in the soil. The Hainich soil is rich in clay, and the clay mineral assemblage includes micas and illites, hydroxy-interlayered vermiculites and smectites, and kaolinite [48]. Due to its mineral assemblage, the site has a high capacity to fix ammonium [49]. Therefore, part of the increase in soil N could be due to ammonium fixation by clay minerals. Larger increases in TN than in OC in deeper soil layers could be due to vertical translocation of N and subsequent sorption, either of organic N or of ammonium, but this cannot be tested with the available data. Separation of organic and inorganic N would be helpful to disentangle the processes controlling soil N retention in future studies.

### Summary and conclusive remarks

The present work supports earlier studies indicating that the old growth forest at the Hainich National park is still accumulating OC in soil. It adds to the growing number of studies showing that soils of temperate forests are currently C sinks. Soil OC increases are estimated to be ∼10% of aboveground C gains. Soil OC and TN gains at the Hainich site seem to be due to increasing litter input, likely because the forest is accumulating biomass, either promoted by N input or due to recovery from historic use. Nitrogen gains in excess of those expected from purely stoichiometric increases in OC indicate that the site is also a sink of deposited inorganic N.

## Supporting Information

Information S1 (DOCX)
