# Supplementary material for: Soil Organic Carbon and Total Nitrogen Gains in an Old Growth Deciduous Forest in Germany
Source: PLoS One. 2014 Feb 20;9(2):e89364. doi: 10.1371/journal.pone.0089364 (PMC3930723; doi:10.1371/journal.pone.0089364)
Supplement: Information S1 — (DOCX) [file pone.0089364.s001.docx]

Supporting information S1

Table 1 Differences in soil properties between 2009 and 2004 (average ± standard error).

| Soil depth | Bulk density | Fine earth | Stone | Root mass | Water content |
| --- | --- | --- | --- | --- | --- |
|  | g cm^-3^ | kg m^-2^ | kg m^-2^ | kg m^-2^ | % |
| 0-5 cm | -0.00 ± 0.03 | -1.06 ± 1.65 | 0.90 ± 0.43 | -0.21 ± 0.12 | 5.10 ± 2.77 |
| 5-10 cm | -0.01 ± 0.04 | -1.87 ± 2.04 | 0.63 ± 0.50 | -0.11 ± 0.09 | 3.82 ± 2.48 |
| 10-20 cm | -0.08 ± 0.03 | -7.92 ± 2.83 | 0.07 ± 1.42 | 0.09 ± 0.25 | 1.64 ± 1.09 |
| 20-30 cm | -0.01 ± 0.04 | -4.99 ± 4.00 | 3.26 ± 3.80 | -0.08 ± 0.12 | 0.82 ± 1.20 |
| 30-40 cm | -0.04 ± 0.04 | -5.61 ± 4.45 | 0.82 ± 5.58 | 0.04 ± 0.09 | 0.57 ± 0.94 |
| 40-50 cm | -0.03 ± 0.04 | 0.64 ± 5.27 | -4.96 ± 8.24 | 0.03 ± 0.09 | -1.11 ± 2.69 |
| 50-60 cm | -0.07 ± 0.08 | -5.08 ± 7.64 | -0.26 ± 10.8 | -0.02 ± 0.15 | -0.18 ± 1.52 |
|  |  |  |  |  |  |
|  | OC concentration | TN concentration | OC-to-TN ratio | OC-stock | N-stock |
|  | g kg^-1^ | g kg^-1^ |  | g m^-2^ | g m^-2^ |
| Oi | -10.3 ± 9.08 | -2.14 ± 0.05 | 6.28 ± 1.85 | -92.0 ± 15.5 | -3.67 ± 0.37 |
| Oe-Oa |  |  |  | -28.4 ± 14.2 | -3.58 ± 0.60 |
| 0-5 cm | 4.33 ± 3.30 | 0.36 ± 0.19 | -0.21 ± 0.21 | 38.3 ± 94.2 | 5.04 ± 6.46 |
| 5-10 cm | 3.18 ± 2.69 | 0.31 ± 0.17 | -0.24 ± 0.26 | 63.8 ± 99.1 | 8.62 ± 6.34 |
| 10-20 cm | 2.16 ± 1.52 | 0.25 ± 0.11 | -0.22 ± 0.21 | 20.0 ± 131 | 7.26 ± 9.85 |
| 20-30 cm | 0.93 ± 0.90 | 0.12 ± 0.07 | -0.19 ± 0.22 | 23.0 ± 115 | 6.80 ± 10.3 |
| 30-40 cm | 1.01 ± 0.88 | 0.12 ± 0.08 | -0.18 ± 0.25 | 46.3 ± 109 | 7.56 ± 9.50 |
| 40-50 cm | 0.51 ± 0.80 | 0.10 ± 0.07 | -0.45 ± 0.30 | 83.4 ± 95 | 12.7 ± 9.17 |
| 50-60 cm | -0.17 ± 1.09 | 0.07 ± 0.09 | -0.36 ± 0.65 | -27.3 ± 132 | 0.27 ± 13.5 |

Table 2 Correlation coefficients for significant (p<0.05) relations between bulk density (BD), fine earth mass per m^-2^ (FE), the stone content (stone), the water content (WC), OC concentrations (OC), total nitrogen concentrations (TN), OC-to-TN ratio (CN), OC stocks (OCst), and TN stocks (TNst). The upper right part of the table shows correlation coefficients for correlations of variables from the 2009 sampling for soil depths from 0-5 to 40-50 cm. The lower left part shows correlation coefficients for relations between the changes of soil variables between 2004 and 2009.

|  |  | BD | Stone | WC | OC | TN | CN | OCst | TNst |
| --- | --- | --- | --- | --- | --- | --- | --- | --- | --- |
| BD | 0-5 5-10 10-20 20-30 30-40 40-50 |  | ns  ns  0.44  0.51  0.67  0.71 | -0.75  -0.32  -0.31  ns  ns  ns | -0.85  -0.67  -0.55  -0.60  -0.62  -0.65 | -0.81  -0.67  -0.41  -0.62  -0.64  -0.70 | -0.69  -0.44  -0.53  -0-35  -0.34  -0.40 | -0.33  -0.25  -0.35  -0.58  -0.63  -0.62 | ns  ns  ns  -0.57  -0.65  -0.65 |
| Stone | 0-5 5-10 10-20 20-30 30-40 40-50 | ns  ns  0.31  0.55  0.67  0.55 |  | ns  ns  ns  0.23  ns  ns | ns  ns  ns  -0.21  -0.38  -0.49 | ns  ns  0.27  ns  -0.40  -0.51 | ns  ns  ns  -0.28  -0.25  -0.34 | ns  ns  -0.28  -0.59  -0.64  -0.68 | -0.25  ns  -0.28  -0.64  -0.71  -0.74 |
| WC | 0-5 5-10 10-20 20-30 30-40 40-50 | -0.71 -0.41 -0.37 ns -0.30 ns | ns  ns  ns  ns  ns  ns |  | 0.78  ns  0.33  ns  ns  ns | 0.73  ns  0.31  ns  ns  ns | 0.73  ns  ns  -0.26  ns  ns | 0.52  ns  ns  -0.23  ns  ns | ns  ns  ns  ns  ns  ns |
| OC | 0-5 5-10 10-20 20-30 30-40 40-50 | -0.72 -0.65 -0.60 -0.34 -0.56 -0.39 | ns  ns  ns  ns  -0.35  ns | 0.68  0.39  0.64  ns  ns  ns |  | 0.95  0.94  0.94  0.99  0.98  0.97 | 0.76  0.74  0.61  0.71  0.74  0.77 | 0.72  0.84  0.81  0.89  0.89  0.95 | 0.31  0.61  0.59  0.84  0.84  0.91 |
| TN | 0-5 5-10 10-20 20-30 30-40 40-50 | -0.69 -0.69 -0.57 -0.32 -0.53 ns | ns  ns  ns  ns  -0.34  ns | 0.62  0.41  0.60  ns  0.27  ns | 0.96  0.95  0.93  0.95  0.96  0.62 |  | 0.60  0.48  0.30  0.58  0.61  0.62 | 0.72  0.77  0.72  0.86  0.86  0.93 | 0.45  0.69  0.62  0.84  0.84  0.93 |
| CN | 0-5 5-10 10-20 20-30 30-40 40-50 | -0.59 -0.40 -0.47 -0.33 -0.29 ns | ns  ns  ns  ns  ns  ns | 0.66  0.26  0.34  ns  -0.27  ns | 0.77  0.79  0.65  0.63  0.51  0.60 | 0.56  0.58  0.35  0.35  0.26  0.28 |  | 0.52  0.66  0.52  0.72  0.70  0.73 | ns  0.23  ns  0.59  0.57  0.60 |
| OCst | 0-5 5-10 10-20 20-30 30-40 40-50 | ns ns -0.23 -0.27 -0.56 -0.30 | -0.42  -0.34  ns  -0.43  -0.58  -0.33 | ns  ns  0.54  ns  ns  ns | 0.54  0.79  0.88  0.89  0.87  0.90 | 0.53  0.74  0.85  0.84  0.83  0.61 | 0.38  0.68  0.49  0.58  0.44  0.47 |  | 0.82  0.88  0.91  0.98  0.98  0.98 |
| TNst | 0-5 5-10 10-20 20-30 30-40 40-50 | ns ns ns -0.26 -0.48 -0.33 | -0.41  -0.39  ns  -0.51  -0.61  -0.37 | ns  ns  0.44  ns  ns  ns | 0.20  0.58  0.71  0.79  0.77  0.81 | 0.33  0.63  0.81  0.84  0.81  0.57 | ns  0.34  ns  0.33  ns  0.24 | 0.89  0.92  0.91  0.95  0.95  0.96 |  |

Table 3 Differences in soil carbon and nitrogen stocks between 2009 and 2004 using average soil masses of the 2004 sampling in each depth interval as reference (average ± standard error).

| Soil mass | OC-stock | N-stock |
| --- | --- | --- |
| kg m^-2^ | g m^-2^ | g m^-2^ |
| 0-39 | 112 ± 115 | 10 ± 6 |
| 39-88 | 73 ± 102 | 10 ± 7 |
| 88-205 | 133 ± 124 | 18 ± 10 |
| 205-332 | 28 ± 110 | 6 ± 9 |
| 332-455 | 53 ± 115 | 9 ± 10 |
| 0-39 | 112 ± 115 | 10 ± 6 |
| 0-88 | 189 ± 200 | 21 ± 12 |
| 0-205 | 323 ± 293 | 39 ± 20 |
| 0-332 | 286 ± 346 | 43 ± 26 |
| 0-455 | 272 ± 384 | 47 ± 29 |
